# Treatment results for hypopharyngeal cancer by different treatment strategies and its secondary primary- an experience in Taiwan

**DOI:** 10.1186/1748-717X-5-91

**Published:** 2010-10-07

**Authors:** Morgan Fu-Ti Chang, Hung-Ming Wang, Chung-Jan Kang, Shiang-Fu Huang, Chien-Yu Lin, Kang-Hsing Fang, Eric Yen-Chao Chen, I-How Chen, Chun-Ta Liao, Joseph Tung-Chieh Chang

**Affiliations:** 1 Department of Radiation Oncology, Hsinchu General Hospital, Hsin-Chu, Taiwan; 2 Division of Hematology/Medical Oncology, Department of Internal Medicine, Chang Gung Memorial Hospital at Linkou, Taoyuan, Taiwan; 3 Department of Otorhinolaryngology/Head and Neck Surgery, Chang Gung Memorial Hospital at Linkou, Taoyuan, Taiwan; 4 Department of Radiation Oncology, Chang Gung Memorial Hospital at Linkou, Taoyuan, Taiwan; 5 Taipei Chang Gung Head and Neck Oncology Group, Chang Gung Memorial Hospital at Linkou, Taoyuan, Taiwan; 6 Department of Medicine, Chang Gung University, Taoyuan, Taiwan; 7 Graduate Institute of Clinical Medical Science, Chang Gung University, Taoyuan, Taiwan

## Abstract

**Purpose:**

The aim of this study was to evaluate treatment results in our hypopharyngeal cancer patients.

**Patients and Methods:**

A total of three hundred and ninety five hypopharyngeal cancer patients received radical treatment at our hospital; 96% were male. The majority were habitual smokers (88%), alcohol drinkers (73%) and/or betel quid chewers (51%). All patients received a CT scan or MRI for tumor staging before treatment. The stage distribution was stage I: 2 (0.5%); stage II: 22 (5.6%); stage III: 57 (14.4%) and stage IV: 314 (79.5%). Radical surgery was used first in 81 patients (20.5%), and the remaining patients (79.5%) received organ preservation-intended treatment (OPIT). In the OPIT group, 46 patients received radiotherapy alone, 156 patients received chemotherapy followed by radiotherapy (CT/RT) and 112 patients received concomitant chemo-radiotherapy (CCRT).

**Results:**

The five-year overall survival rates for stages I/II, III and IV were 49.5%, 47.4% and 18.6%, respectively. There was no significant difference in overall and disease-specific survival rates between patients who received radical surgery first and those who received OPIT. In the OPIT group, CCRT tended to preserve the larynx better (p = 0.088), with three-year larynx preservation rates of 44.8% for CCRT and 27.2% for CT/RT. Thirty-seven patients developed a second malignancy, with an annual incidence of 4.6%.

**Conclusions:**

There was no survival difference between OPIT and radical surgery in hypopharyngeal cancer patients at our hospital. CCRT may offer better laryngeal preservation than RT alone or CT/RT. However, prospective studies are still needed to confirm this finding. Additionally, second primary cancers are another important issue for hypopharyngeal cancer management.

## Introduction

Patients with carcinoma of the hypopharynx frequently have advanced disease at the time of presentation. These patients have some of the worst prognoses of all head and neck cancer patients, and combined-modality therapy is usually required to achieve a cure. The conventional treatment for advanced, but resectable, cases has been surgery followed by post-operative adjuvant therapy, and five-year survival rates vary from 10% to 60% [1-5]. Recently, the integration of chemotherapy and radiotherapy was investigated for organ preservation in patients with locally advanced hypopharyngeal cancers. The results of these prospective trials were encouraging; they indicated that the larynx could be preserved using combined chemotherapy and radiotherapy without compromising overall survival rates [6-10].

Two phase III trials [11,12] of sequential chemotherapy and radiotherapy for resectable laryngeal or hypopharyngeal cancer revealed survival rates similar to those achieved with surgery and post-operative irradiation, but the larynx was preserved for many patients in the former group. On the other hand, a meta-analysis [13] of six trials comparing induction chemotherapy and radiotherapy with alternating or concomitant chemo-radiotherapy (CCRT) revealed a hazard ratio of 0.91 (0.79-1.06) in favor of the latter. This analysis also showed a five-year survival benefit of 32%- 40% when chemotherapy was added concomitantly to radiotherapy. Growing evidence suggests that CCRT may improve loco-regional tumor control in locally advanced head and neck cancers and, more importantly, improve survival rates compared with the sequential regimen or radiotherapy alone [14,15].

To the best of our knowledge, no existing data demonstrate whether CCRT could enhance organ preservation in hypopharyngeal cancer patients. In this article, we present treatment results for our hypopharyngeal cancer patients. Furthermore, we determine whether concomitant use of chemotherapy offers the best chance of organ preservation.

## Patients and Methods

From January 1994 to May 2004, 430 hypopharyngeal cancer patients were referred for radiotherapy evaluation. We excluded 35 patients who refused radical therapy, leaving 395 patients for analysis. All patients received computed tomography scans or magnetic resonance imaging (MRI) for staging prior to radical treatment. Initially, 81 patients (20.5%) first received radical surgery, and the remaining patients (79.5%) underwent organ preservation-intended therapy (OPIT). Treatment decisions were based on the preference of the serving physician and/or patient. In the group that initially received radical surgery, patients with risk factors such as positive pathological margin, more than two lymph node metastases or extracapsular extension of the lymph nodes also received concomitant chemotherapy when post-operative radiotherapy was performed. In the OPIT group, 47 patients received radiotherapy (RT) alone, 188 patients received induction chemotherapy followed by radiotherapy (CT/RT) and 79 patients received CCRT.

The chemotherapy (CT) regimen, PTL, was detailed in our previous report [16]. In brief, it consists of 50 mg/m^2 ^cisplatin (P) on Day 1, followed by 800 mg/m^2 ^oral tegafur (T) per day and 60 mg oral leucovorin (L) per day for 14 days. The CT was administered at outpatient clinics in 14-day cycles. In the CT/RT group, re-evaluation after three cycles of chemotherapy led to the termination of CT if tumor responses were less than partial responses. Otherwise, PTL regimens were continued for up to six cycles before radiotherapy. Patients achieving at least good partial responses at the primary site after neoadjuvant chemotherapy received radiotherapy or chemo-radiotherapy for organ preservation.

Radiotherapy was performed by three-field technique; it consisted of conventional bilateral opposing fields with a matching anterior lower neck portal. The daily fractionation size was 1.8 or 2 Gy, with five fractions per week. The median dose to the gross tumor volume was 68.4 Gy (range: 60-76 Gy), and to clinical target volume was 45 Gy (range 45-46 Gy). The planning target volume was created by adding 5-7 mm margin from clinical target volume. For the group receiving radical surgery first, the post-operative radiotherapy dose was 60-68.4 Gy, depending on the pathology risk factor; for the OPIT group, the dose range was 68.4-76 Gy. The spinal cord was shielded by customerized cerrobend block or multi-leaf collimator after 45-46 Gy and the posterior neck regions were boosted with a 9-12-MeV electron beam for an additional 14-24 Gy in 7-12 fractions, according to the status of the regional lymph nodes.

In the organ preservation group, planned neck dissection was not routinely performed. Salvage surgery or neck dissection was undertaken when any residual lesion was noted in the post-treatment evaluation, which was usually performed three months after radical treatment or in the case of tumor progression.

All patients were followed in the clinic every one to two months for the first two years, and then every three to four months in the third to fifth years. Computer tomography scans, bone scans, chest X-rays, SMA and CBC were scheduled routinely (at least annually) for at least the first three years post-treatment to detect recurrence. The primary endpoint of our study was overall survival rate, with a second endpoint of disease-specific survival rate (DSS). The duration of survival was defined as the time from the first date of radical treatment to the date of the event, which was death for the overall survival rate or tumor-related mortality for DSS. For survival with a preserved larynx (OSP), the event was defined as death or total laryngopharyngectomy. Loco-regional or distant control meant that no recurrence could be verified by pathological examination or progressive changes in serial image studies when no tissue proof was available. Statistical Package for the Social Sciences software (SPSS Inc., Chicago, IL) was used for statistical analysis. The Kaplan-Meier method was used to estimate survival rates with the log-rank test for subgroup analyses. A p-value of < 0.05 was considered significant. Multivariate analyses were assessed using the Cox-regression model.

## Results

### Patient population

The characteristics of all patients are listed in Table 1. Ninety-six percent were male, and the median age was 56 years (range: 15-87). The majority of patients were habitual smokers (86.6%), alcohol drinkers (69.6%) and/or betel quid chewers (47.1%). All patients were re-staged according to the AJCC 2002 staging system. The stage distribution was as follows: stage I: 2 (0.5%), stage II: 22 (5.6%), stage III: 57(14.4%) and stage IV: 314 (79.5%).

**Table 1:** Patient characteristics

	Case Numbers (percentage)	Radical surgery group	Organ preservation group	P-value
Age, years				0.035
≦55	188 (47.6%)	47	141	
> 55	207 (52.4%)	34	173	
Gender				0.176
Male	380 (96.2%)	80	300	
Female	15 (3.8%)	1	14	
Smoking				0.856
Yes	342 (86.6%)	71	271	
No	53 (13.4%)	10	43	
Alcohol drinking				0.869
Yes	275 (69.6%)	57	218	
No	120 (30.4%)	24	96	
Betel nut chewing				
Yes	186 (47.1%)	41	145	0.533
No	209 (52.9%)	40	169	
T stage				0.012
T1	19 (4.8%)	4	15	
T2	71 (18%)	11	60	
T3	73 (18.5%)	6	63	
T4	232 (58.7%)	60	172	
N stage				0.300
N0	113 (28.6%)	20	93	
N1	73 (18.5%)	12	61	
N2	154 (39%)	39	115	
N3	55 (13.9%)	10	45	
Overall Stage				0.013
I	2 (0.5%)	0	2	
II	22 (5.6%)	2	20	
III	57 (14.4%)	4	53	
IV	314(79.5%)	75	239	

### Overall survival and disease-specific survival

The median follow-up time was 5.09 years. At the time of analysis, 269 patients had died: of these, 185 died of local disease, 35 died of distant metastasis and 49 died of a second primary tumor or other intercurrent disease. The five-year overall survival rate for all patients was 24.8%. The five-year overall survival rates for stages I/II, III and IV were 49.5%, 47.4% and 18.6%, respectively (p < 0.001). The five-year DSS rates for stages I/II, III and IV were 67.4%, 53.5% and 25.5%, respectively (p < 0.001). The results of subgroup analyses are illustrated in Table 2.

**Table 2:** Prognostic factors for survival rates, univariate analysis

	Numbers (n)	5-yr OS rate (%)	p-value	5-yr DSS rate (%)	p-value
Age, years-old			0.747		0.961
≦55	188	25.5		35.2	
> 55	207	24.4		31.4	
Smoking			0.029		0.075
Yes	342	22.5		30.3	
No	53	41.7		49.8	
Alcohol drinking			0.081		0.158
Yes	275	22.6		30.8	
No	120	29.9		37.3	
Betel nut chewing			0.360		0.159
Yes	186	24.4		32.2	
No	209	25.0		31.3	
T-stage			< 0.001		< 0.001
T1	19	54.3		68.6	
T2	71	38.1		45.1	
T3	69	30.7		38.2	
T4	232	17.2		24.4	
N-stage			< 0.001		< 0.001
N0	113	32.4		40.6	
N1	73	36.4		43.6	
N2	154	20.6		30.1	
N3	55	0		0	
Stage			< 0.001		< 0.001
I/II	24	49.5		67.4	
III	57	47.4		53.5	
IV	314	18.6		25.5	
Treatment			0.229		0.069
Radical surgery first	81	18.8		24.2	
Organ preservation	314	27.0		35.9	

**Figure 1:**
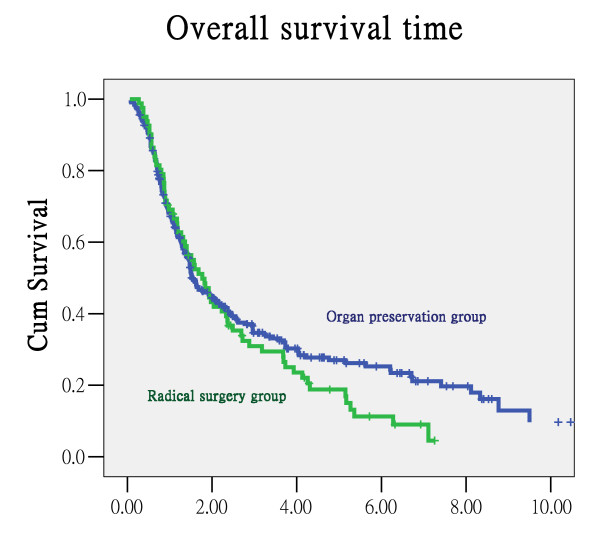
**Overall survival curve**.

**Figure 2:**
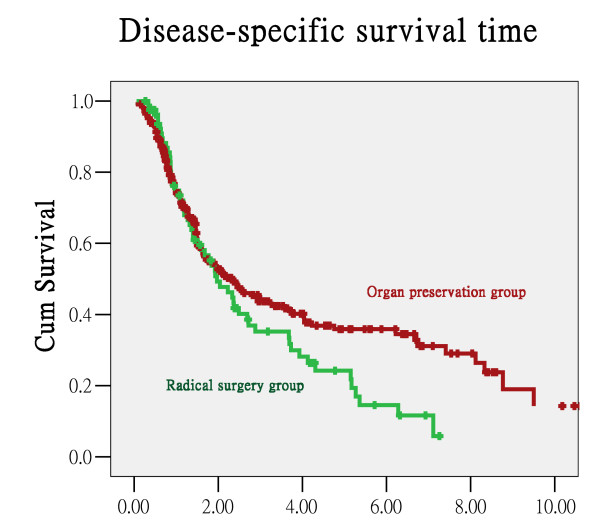
**Disease-specific survival curve**.

There was no significant difference in the overall survival rate or DSS rate between the group of patients receiving radical surgery first and the organ-preservation intended treatment group. The five-year overall survival rate and DSS rate were 18.8% and 24.2% in the radical surgery-first group and 27% and 35.9% in the OPIT group, respectively (Figure 1 &2). There was no significant difference in the survival rate based on the type of combination between chemotherapy and radiotherapy. The five-year overall survival rate and DSS rate were 20.5% and 29.2% for the CT/RT group and 43.1% and 53% for the CCRT group, respectively (p = 0.200 for overall survival rate and p = 0.397 for DSS). Besides, when confine the patients into stage III and IV, there is no significant difference between OPIT group and radical surgery group in overall survival rates and disease-free survival rates (p-value = 0.449 and 0.427 respectively).

The five-year overall survival rate was 45.9% and the DSS rate was 54.4% in patients without evidence of recurrence. Recurrent patients who suffered from locoregional failures had better prognoses than those with distant failures (Table 2). T-stage, N-stage and recurrence were all independent predictors of overall survival and DSS after multivariate analysis (Table 3).

**Table 3:** Multivariate analysis

	T-stage		N-stage		Recurrence	
	p-value	Hazard ratio (95% CI)	p-value	Hazard ratio (95% CI)	p-value	Hazard ratio (95% CI)
5-yr overall survival rate	< 0.001	0.332 (0.169-0.652)	< 0.001	0.321 (0.218-0.470)	0.013	0.503 (0.32-0.790)
5-yr disease-specific survival rate	0.003	0.325 (0.151-0.699)	< 0.001	0.290 (0.189-0.445)	0.004	0.435 (0.264-0.717)

For patients who only experienced loco-regional recurrences, salvage surgery with or without adjuvant radiotherapy and chemotherapy was given under certain conditions. The five-year DSS rate was 27.8%, and the overall survival rate was 19.6%. Chemotherapy was given to patients with distant metastasis with or without loco-regional control and good performance status, and to patients with supportive care but with poor performance status. However, none of these patients survived longer than three years. The median survival time for patients with distant metastasis and without loco-regional control was 1.4 years; patients with recurrence at both distant and loco-regional sites survived for an average of 1.19 years.

### Organ preservation

**Figure 3:**
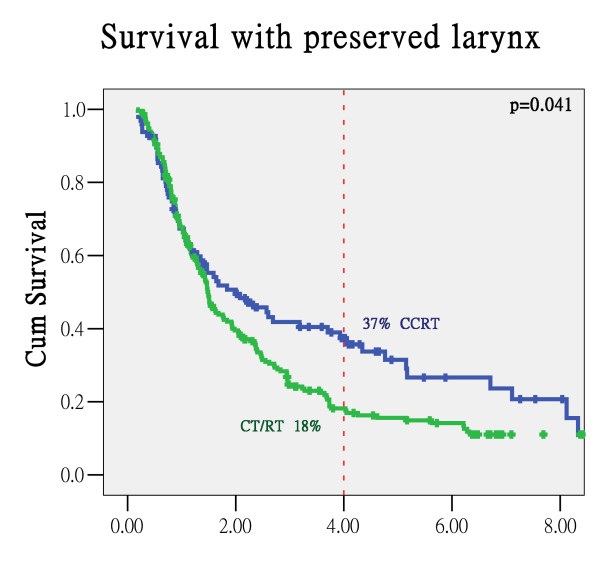
**Survival with larynx preservation curve in the organ preservation group**.

In the organ preservation group, 93 patients (29.6%) survived with a preserved larynx at three years. There were no significant differences in patient characteristics between C/T+RT and CCRT except for less betel nut use in CCRT patients. Patients in early T-stage or N-stage had higher rates of larynx preservation. Smoking, alcohol drinking or betel quid chewing were not important factors for organ preservation. However, patients who received concomitant chemotherapy had a higher chance of survival with a preserved larynx when compared with patients who received induction chemotherapy (CT/RT; 37% vs. 18% of 4-year OSP, p = 0.041; Figure 3).

### Second primary malignancy

**Figure 4:**
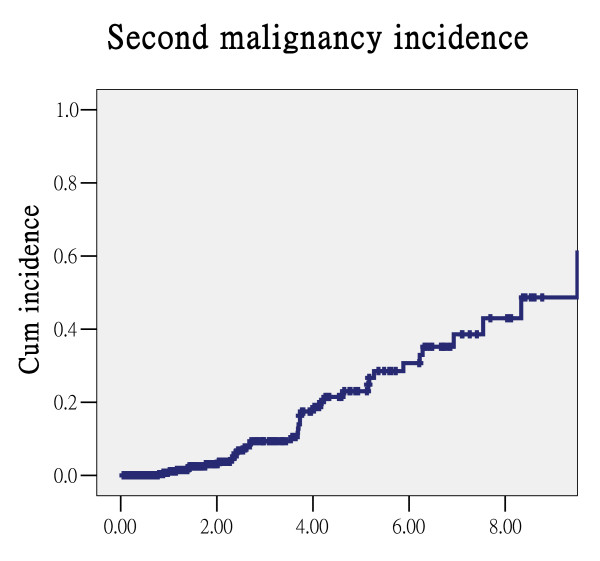
**Cumulative incidence of second malignancy**.

During follow-up, 37 patients experienced a second primary malignancy. There were sixteen head and neck cancers (five tongue, four oropharynx, three mouth floor, two buccal region, one larynx and one submandibular gland), twelve esophageal cancers, twelve lung cancers, six bladder cancers and one colon cancer. The median time to the development of the second primary malignancy was 2.64 years, with a 4.6% rate of annual incidence (Figure 4).

## Discussion

Symptoms of hypopharyngeal cancers occur late, so most of them are diagnosed at an advanced stage. Almost 80% of our patients presented with stage IV disease. Among head and neck cancers, hypopharyngeal cancer has the worst prognosis. The five-year overall survival rate was 24.8% in our series, which is comparable to results from other studies where overall survival rates varied from 10 to 60% [1-3,6-10,12,17-23].

The conventional treatment for locally advanced but resectable head and neck cancers has been surgery with post-operative adjuvant therapy depending on the risk factors for recurrence after surgery. Radiotherapy, however, is the treatment of choice for unresectable or medically inoperable patients. To improve survival rates and preserve organs, a combination of chemotherapy and radiotherapy was introduced. Most retrospective studies of head and neck cancers included various subsites (Table 4). Some series revealed a significant rate of organ preservation with similar survival rates between surgery and chemo-radiotherapy in head and neck cancer patients [1,4,6-12,15,16,18,19,23-27], especially for laryngeal cancer. In this study, we separated the entire patient population into two main treatment groups: radical surgery or organ preservation. There was no significant difference in the overall survival rate and DSS rate between patients who received radical surgery first and patients in the organ preservation group. However, patients who survived longer than three years had a 33.2% larynx preservation rate in the latter group.

**Table 4:** Organ preservation studies of head-and-neck cancers

Author	Year of collection	Case number	Cancer subsite	Treatment	Survival rate	Organ preservation rate
**VALCSG **[11]		332	Stage III/IV LAx	Surgery	68% at 2 yr	
				Induction C/T + RT	68% at 2 yr	64% at 2 yr
**Malone et al. **[25]	1993-2000	40	Stage III/IV BOT	OP+adj-CCRT	74.7% at 2 yr	-
**Sewnaik et al. **[5]	1985-1994	893	HPx	Surgery and RT	32% at 5 yr	
**Adelstein et al. **[24,24]	1989-2002	222	All head and neck	CCRT	65.7% at 5 yr	62.2% at 5 yr
**Soo et al. **[4]		119	All head and neck	Surgery	50% at 3 yr^#^	
				CCRT	40% at 3 yr^#^	45% at 3 yr
**Hanna et al. **[7]	1996-2002	127	OPx, LAx, HPx, OC	CCRT	57% at 3 yr	-
**Urba et al. **[6]		59	BOT, HPx	Induction C/T + CCRT	64% at 3 yr	52% at 3 yr
**Current series**	1994-2004	395	HPx	Surgery	18.8% at 5 yr	
				CCRT	27% at 5 yr	44.8% at 3 yr 37% at 4 yr

#: disease-free survival; Lax: larynx; BOT: base of tongue; HPx: hypopharynx; OPx: oropharynx; OC: oral cavity

Two large phase III randomized trials demonstrated that induction chemotherapy followed by definite radiotherapy (CT/RT) yielded survival rates similar to those in patients receiving surgery and irradiation for laryngeal and pyriform sinus cancer, respectively [11,12]. The rationale for using induction chemotherapy is the identification of patients for radiotherapy according to the high predictability of subsequent radiotherapy response based on the response to chemotherapy. Therefore, induction chemotherapy could be used as a surrogate for patient selection to identify patients who are eligible for organ preservation. This procedure could avoid the inevitable severe complications for patients who receive high-dose RT followed by salvage surgery.

However, the results of a recent RTOG study of laryngeal cancer patients [11] challenged the role of induction chemotherapy in selecting the "right" patients for organ preservation. Concomitant chemo-radiotherapy can achieve better rates of organ preservation than induction chemotherapy selection followed by radiotherapy. Furthermore, in this study, eleven patients selected for radical surgery due to a poor response to induction chemotherapy did not accept radical surgery, so they received chemotherapy and radiotherapy. All of these patients achieved complete remission after radical treatment and, consequently, only one patient required a laryngectomy. Although the number is small and there may be some bias in the patients' treatment choices, the use of induction chemotherapy as a predictor of organ preservation needs further study, especially in an era where more patients are choosing CCRT.

Concomitant chemotherapy may contribute to the radiosensitizing effect of radiotherapy and thus improve tumor control. A large meta-analysis showed that the survival rate increased significantly when chemotherapy was added to the treatment of head and neck cancers [13]. Although the heterogeneity of these 63 trials (including 10741 patients) limited the identification of conclusive results, chemotherapy given concomitantly with radiotherapy still had substantial benefits, corresponding to an absolute five-year survival benefit of **8%**. Our study also found that patients who received CCRT had higher rates of survival with larynx preservation (44.8% at three years). Although there was no significant difference in overall survival, the use of CCRT allows the possibility of larynx preservation, which may have an impact on a patient's social activity and quality of life.

In retrospective trials of radiotherapy versus surgery, there is always the possibility of strong selection bias: usually the surgeons get the "better" patients because their patients need to be operable and/or resectable. In this study, a similar bias may have occurred. However, the OPIT group did not show a worse tumor control or survival rate than surgical group, and some large unresectable tumors were included in the OPIT group. Prospective studies would be valuable in addressing these issues.

Most patients in our study relapsed at loco-regional sites, and their five-year overall survival rate was only 19.6%, which suggests that conventional radiotherapy techniques using bilateral opposing fields may compromise radiation dose coverage of the target after blocking of the spinal cord at doses of 46-50 Gy. Some studies of recent modern radiotherapy techniques such as intensity-modulated radiotherapy (IMRT) with concomitant chemotherapy yielded promising loco-regional control rates as well as disease-free and overall survival rates for hypopharyngeal cancer [2,28,29]. Some studies also revealed that it is possible to decrease the severity of late toxicities such as dysphagia and aspiration using IMRT to spare the larynx and swallowing muscles [30,31].

Second primary cancers were a major cause of death in this study, with an annual incidence rate of 4.6%. The median time to the development of a second primary malignancy was 2.64 years. This incidence is similar to that reported in our previous study on oral cavity cancer [16], but the occurrence sites are slightly different. In oral cavity cancer, the most common second primary area of occurrence is the head and neck region, especially the oral cavity area (70.3%). However, in this study, about 60% (21/37) of cancers occurred below the clavicle despite all of the patients having similar habits of betel quid chewing, smoking and/or alcohol drinking. Squamous cell carcinoma of upper aero-digestive tract (including oral cavity, pharynx, esophagus and lung) is the most common cancer that occurs in Taiwanese man, and the incidence of oral cavity cancer and esophageal cancer is increasing 13.1% and 4.1% respectively in ten years in Taiwan[32]. On the other hand, most of our patients have the habits of smoking, betel quid chewing and alcohol consumption, and the concept of field cancerization from Slaughter et al. [33] may explain the relative high incidence of second primary malignancy in our patients.

## Conclusion

The majority of our hypopharyngeal cancer patients presented at stage IV. There was no survival difference between the organ preservation intended therapy and radical surgery groups. Patients who received CCRT had a better chance of survival with a preserved larynx compared with patients who received induction chemotherapy. Secondary cancer was a major cause of death. The median time to the development of a second primary malignancy was 2.64 years, with a 4.6% annual incidence. We suggest that organ preservation intended therapy, especially CCRT, should be considered first for patients with advanced hypopharyngeal cancer patients who refuse, or are unable to undergo, radical surgery.

## Competing interests

The authors declare that they have no competing interests.

## Authors' contributions

MFC and JTC designed and coordinated the study. Patient accrual and clinical data collection was done by all authors. Data analysis and treatment data collection was done by MFC and JTC. MFC prepared the manuscript.

HW and JTC revised critically for important intellectual content. All authors read and approved the final manuscript.

## References

[B1] Lajtman Z Manestar D A comparison of surgery and radiotherapy in the management of advanced pyriform fossa carcinoma Clin Otolaryngol Allied Sci 2001 26 59 61 10.1046/j.1365-2273.2001.00430.x 11298170

[B2] Lee NY O'Meara W Chan K Della-Bianca C Mechalakos JG Zhung J Wolden SL Narayana A Kraus D Shah JP Pfister DG Concurrent chemotherapy and intensity-modulated radiotherapy for locoregionally advanced laryngeal and hypopharyngeal cancers Int J Radiat Oncol Biol Phys 2007 69 459 468 17493769 10.1016/j.ijrobp.2007.03.013

[B3] Arriagada R Eschwege F Cachin Y Richard JM The value of combining radiotherapy with surgery in the treatment of hypopharyngeal and laryngeal cancers Cancer 1983 51 1819 1825 10.1002/1097-0142(19830515)51:10<1819::AID-CNCR2820511011>3.0.CO;2-G 6831347

[B4] Soo KC Tan EH Wee J Lim D Tai BC Khoo ML Goh C Leong SS Tan T Fong KW Surgery and adjuvant radiotherapy vs concurrent chemoradiotherapy in stage III/IV nonmetastatic squamous cell head and neck cancer: a randomised comparison Br J Cancer 2005 93 279 286 10.1038/sj.bjc.6602696 16012523 PMC2361563

[B5] Sewnaik A Hoorweg JJ Knegt PP Wieringa MH van der Beek JM Kerrebijn JD Treatment of hypopharyngeal carcinoma: analysis of nationwide study in the Netherlands over a 10-year period Clin Otolaryngol 2005 30 52 57 10.1111/j.1365-2273.2004.00913.x 15748191

[B6] Urba SG Moon J Giri PG Adelstein DJ Hanna E Yoo GH Leblanc M Ensley JF Schuller DE Organ preservation for advanced resectable cancer of the base of tongue and hypopharynx: a Southwest Oncology Group Trial J Clin Oncol 2005 23 88 95 10.1200/JCO.2005.04.017 15625363

[B7] Hanna E Alexiou M Morgan J Badley J Maddox AM Penagaricano J Fan CY Breau R Suen J Intensive chemoradiotherapy as a primary treatment for organ preservation in patients with advanced cancer of the head and neck: efficacy, toxic effects, and limitations Arch Otolaryngol Head Neck Surg 2004 130 861 867 10.1001/archotol.130.7.861 15262764

[B8] Robbins KT Fontanesi J Wong FS Vicario D Seagren S Kumar P Weisman R Pellitteri P Thomas JR Flick P A novel organ preservation protocol for advanced carcinoma of the larynx and pharynx Arch Otolaryngol Head Neck Surg 1996 122 853 857 8703389 10.1001/archotol.1996.01890200043010

[B9] Rudat V Pfreundner L Hoppe F Dietz A Approaches to preserve larynx function in locally advanced laryngeal and hypopharyngeal cancer Onkologie 2004 27 368 375 10.1159/000079090 15347892

[B10] Zelefsky MJ Kraus DH Pfister DG Raben A Shah JP Strong EW Spiro RH Bosl GJ Harrison LB Combined chemotherapy and radiotherapy versus surgery and postoperative radiotherapy for advanced hypopharyngeal cancer Head Neck 1996 18 405 411 10.1002/(SICI)1097-0347(199609/10)18:5<405::AID-HED3>3.0.CO;2-9 8864731

[B11] Induction chemotherapy plus radiation compared with surgery plus radiation in patients with advanced laryngeal cancer. The Department of Veterans Affairs Laryngeal Cancer Study Group N Engl J Med 1991 324 1685 1690 10.1056/NEJM199106133242402 2034244

[B12] Lefebvre JL Chevalier D Luboinski B Kirkpatrick A Collette L Sahmoud T Larynx preservation in pyriform sinus cancer: preliminary results of a European Organization for Research and Treatment of Cancer phase III trial. EORTC Head and Neck Cancer Cooperative Group J Natl Cancer Inst 1996 88 890 899 10.1093/jnci/88.13.890 8656441

[B13] Pignon JP Bourhis J Domenge C Designe L Chemotherapy added to locoregional treatment for head and neck squamous-cell carcinoma: three meta-analyses of updated individual data. MACH-NC Collaborative Group. Meta-Analysis of Chemotherapy on Head and Neck Cancer Lancet 2000 355 949 955 10768432

[B14] Adelstein DJ Sharan VM Earle AS Shah AC Vlastou C Haria CD Damm C Carter SG Hines JD Simultaneous versus sequential combined technique therapy for squamous cell head and neck cancer Cancer 1990 65 1685 1691 10.1002/1097-0142(19900415)65:8<1685::AID-CNCR2820650804>3.0.CO;2-S 2317751

[B15] Calais G Alfonsi M Bardet E Sire C Germain T Bergerot P Rhein B Tortochaux J Oudinot P Bertrand P Randomized trial of radiation therapy versus concomitant chemotherapy and radiation therapy for advanced-stage oropharynx carcinoma J Natl Cancer Inst 1999 91 2081 2086 10.1093/jnci/91.24.2081 10601378

[B16] Wang HM Wang CS Chen JS Chen IH Liao CT Chang TC Cisplatin, tegafur, and leucovorin: a moderately effective and minimally toxic outpatient neoadjuvant chemotherapy for locally advanced squamous cell carcinoma of the head and neck Cancer 2002 94 2989 2995 10.1002/cncr.10570 12115388

[B17] Cachin Y Eschwege F Combination of radiotherapy and surgery in the treatment of head and neck cancers Cancer Treat Rev 1975 2 177 191 10.1016/S0305-7372(75)80002-8 1104162

[B18] Featherstone CJ Clarke S Jackson MA Shannon KF McNeil EB Tin MM Clifford A O'Brien CJ Treatment of advanced cancer of the larynx and hypopharynx with chemoradiation ANZ J Surg 2004 74 554 558 10.1111/j.1445-2197.2004.03056.x 15230789

[B19] Garden AS Harris J Vokes EE Forastiere AA Ridge JA Jones C Horwitz EM Glisson BS Nabell L Cooper JS Preliminary results of Radiation Therapy Oncology Group 97-03: a randomized phase ii trial of concurrent radiation and chemotherapy for advanced squamous cell carcinomas of the head and neck J Clin Oncol 2004 22 2856 2864 10.1200/JCO.2004.12.012 15254053

[B20] Kim JG Sohn SK Kim DH Baek JH Jeon SB Chae YS Lee KB Park JS Sohn JH Kim JC Park IK Phase II study of concurrent chemoradiotherapy with capecitabine and cisplatin in patients with locally advanced squamous cell carcinoma of the head and neck Br J Cancer 2005 93 1117 1121 10.1038/sj.bjc.6602849 16251869 PMC2361495

[B21] Lavertu P Adelstein DJ Saxton JP Secic M Eliachar I Strome M Larto MA Wood BG Aggressive concurrent chemoradiotherapy for squamous cell head and neck cancer: an 8-year single-institution experience Arch Otolaryngol Head Neck Surg 1999 125 142 148 10037279 10.1001/archotol.125.2.142

[B22] Razack MS Sako K Marchetta FC Calamel P Bakamjian V Shedd DP Carcinoma of the hypopharynx: success and failure Am J Surg 1977 134 489 491 10.1016/0002-9610(77)90383-X 911032

[B23] Rudat V Wannenmacher M Role of multimodal treatment in oropharynx, larynx, and hypopharynx cancer Semin Surg Oncol 2001 20 66 74 10.1002/ssu.1018 11291134

[B24] Adelstein DJ Saxton JP Rybicki LA Esclamado RM Wood BG Strome M Lavertu P Lorenz RR Carroll MA Multiagent concurrent chemoradiotherapy for locoregionally advanced squamous cell head and neck cancer: mature results from a single institution J Clin Oncol 2006 24 1064 1071 10.1200/JCO.2005.01.5867 16505425

[B25] Malone JP Stephens JA Grecula JC Rhoades CA Ghaheri BA Schuller DE Disease control, survival, and functional outcome after multimodal treatment for advanced-stage tongue base cancer Head Neck 2004 26 561 572 10.1002/hed.20012 15229898

[B26] Ghi MG Paccagnella A D'Amanzo P Mione CA Fasan S Paro S Mastromauro C Carnuccio R Turcato G Gatti C Neoadjuvant docetaxel, cisplatin, 5-fluorouracil before concurrent chemoradiotherapy in locally advanced squamous cell carcinoma of the head and neck versus concomitant chemoradiotherapy: a phase II feasibility study Int J Radiat Oncol Biol Phys 2004 59 481 487 15145166 10.1016/j.ijrobp.2003.10.055

[B27] Forastiere AA Goepfert H Maor M Pajak TF Weber R Morrison W Glisson B Trotti A Ridge JA Chao C Concurrent chemotherapy and radiotherapy for organ preservation in advanced laryngeal cancer N Engl J Med 2003 349 2091 2098 10.1056/NEJMoa031317 14645636

[B28] Studer G Lutolf UM Davis JB Glanzmann C IMRT in hypopharyngeal tumors Strahlenther Onkol 2006 182 331 335 10.1007/s00066-006-1556-2 16703288

[B29] Studer G Peponi E Kloeck S Dossenbach T Huber G Glanzmann C Surviving hypopharynx-larynx carcinoma in the era of IMRT Int J Radiat Oncol Biol Phys 77 1391 1396 20056352 10.1016/j.ijrobp.2009.07.005

[B30] Eisbruch A Schwartz M Rasch C Vineberg K Damen E Van As CJ Marsh R Pameijer FA Balm AJ Dysphagia and aspiration after chemoradiotherapy for head-and-neck cancer: which anatomic structures are affected and can they be spared by IMRT? Int J Radiat Oncol Biol Phys 2004 60 1425 1439 15590174 10.1016/j.ijrobp.2004.05.050

[B31] Carrara-de Angelis E Feher O Barros AP Nishimoto IN Kowalski LP Voice and swallowing in patients enrolled in a larynx preservation trial Arch Otolaryngol Head Neck Surg 2003 129 733 738 10.1001/archotol.129.7.733 12874074

[B32] Department of Health the Executive Yuan, February 2010. Cancer registry: annual report in Taiwan area in 2007

[B33] Slaughter DP Southwick HW Smejkal W Field cancerization in oral stratified squamous epithelium; clinical implications of multicentric origin Cancer 1953 6 963 968 10.1002/1097-0142(195309)6:5<963::AID-CNCR2820060515>3.0.CO;2-Q 13094644

